# Influence of the use of social media on patients changing dental practice: a web-based questionnaire study

**DOI:** 10.1186/s12903-023-03078-9

**Published:** 2023-06-06

**Authors:** Yolanda Freire, Margarita Gómez Sánchez, Ana Suárez, Gerardo Joves, Marlen Nowak, Víctor Díaz-Flores García

**Affiliations:** Department of Preclinical Dentistry, School of Biomedical Sciences, Universidad Europea de Madrid, Madrid, Spain

**Keywords:** Social media, Dentistry, Dental practices, Patient behavior

## Abstract

**Background:**

Recent years have seen an increase in the use of social media, by the healthcare sector, including dentistry. Indeed, social media may now be important communication tools for dental practices and patients.

This work analyzes the influence of patient (male and female) use of dental practice social media on their decision to change practice. The results also provide insight into the factors patients considered important when choosing a dental practice.

**Methods:**

This study was approved by the Ethics Committee of the Universidad Europea de Madrid (No. CIPI/22.022). Using a web-based questionnaire, a cross-sectional study was undertaken involving a Spanish population that made use of dental services. The questionnaire consisted of four parts covering provision of informed consent, the collection of sociodemographic data, patient use of dental practice social media, and the factors considered important when changing dental practice.

**Results:**

All participants gave their informed consent regarding inclusion. No compensation was provided for participation. 588 people responded to the questionnaire, of whom 503 were eligible for inclusion. Most respondents were female (312/503; 62%). Most (151/503; 30%) last changed dental practice during the previous 2–5 years. 208/503 (41.4%) indicated that they had visited dental practice social media. 118/503 (23.5%) indicated that they had made use of such the last time they changed dental practice; 102 of these 118 (85.6%) reported their experience to have influenced their decision to change. Respondents who had changed practice in the last 5 years had interacted with dental practice social media more than those who changed over 11 years ago (*p* < .05), and those who changed practice in the present/past year were more influenced by these media (*p* < .05). The factor considered most important was 'Facilities and technology'. No gender-based differences were seen for any measured variable (*p* < .05).

**Conclusions:**

Different factors affect the selection of a new dental practice, but respondents who changed practice in the last few years were more likely to have made use of dental practice social media, which, for some, influenced their final decision to change. Dental practices might do well to consider using social media as communication and marketing tools.

## Background

The Internet has become an important part of everyday life, its use often eclipsing that of traditional communication media [1]. Different definitions of social media exist, although the consensus in the literature is that social media enables people to exchange information [2, 3]. The Cambridge Dictionary defines social media as “websites and computer programs that allow people to communicate and share information on the internet using a computer or mobile phone” [4]. In 2021, 92% of households in the European Union (EU) had Internet access, an increase of 26 points compared to 2011 [5]. Different social media sites have different features and uses [6]. Facebook (Meta Platforms, Inc, CA, USA) allows communication via posts, photographs, chats and personal messages [7]; it is one of the most popular platforms [8] with more than 2.23 million monthly users [9]. YouTube (YouTube, LLC, San Brunco, CA, USA), with more than a billion users daily [10], offers videos for free viewing [11, 12], and video-uploading by anyone with an account [11]. Twitter (Twitter Inc., San Francisco, CA, USA) is a text-focused medium [13] that enables the sharing of information and discussion on almost any topic [14]. Instagram (owned by Meta Platforms, Inc.) offers live-stream videos [13] and the ability to share photographs and videos [15]. LinkedIn (Microsoft Corporation, Redmond, WA, USA) is the most popular platform for professional use [6], allowing achievements to be shared [10]. In 2021, 57% of people in the EU aged 16–74 used the internet for social networking, involving the use of sites such as Facebook, Instagram and Twitter [5].

The Internet has also become a source of health information [11, 12, 16]. In 2019, 53% of EU citizens between 16 and 74 years of age sought health information [17]. YouTube is one of the platforms of choice for this activity [12]; it offers a large amount of such information [18]. Social media also act as a means of communication for colleagues, patients and the public on health issues [19]. They can also be used by many public health professionals and organizations for other purposes such as health education, telemedicine [19], social marketing [20], scientific research, recruitment, career development, and professional networking [19]. Indeed, health professionals now make great use of social media [21, 22].

These media are becoming increasingly important for dentistry [16, 23], with many dentists, particularly younger ones, making use of them [24]. One use is related to health promotion [19]. For example, pediatric dentists use these media in patient education programs [3] and to discuss anonymized cases [19]. They can also be used in career development by allowing contact between practitioners otherwise unlikely to meet [2]. They are also used for teledentistry, dental educational activities [19], and to obtain information on prospective employees [25]. They can also be used by dental patients. For example, patients have shared their testimonies about dental implants on YouTube [26], and their experience of dental pain [27] and orthodontic treatment on Twitter [28].

Social media also provide a means of communication between patient and dentists. Sivrikaya et al. [16] examined the effects of dentist-patient communication on dental anxiety and observed that communication via Instagram prior to an intervention reduced it. Henry et al. [25] reported that the marketing strategies most employed by orthodontic clinics involved the use of social media and a website; both types of platform were positively related to the acquisition of new patients. Cox et al. [7] reported that many consumers were likely to visit a clinic's website and Facebook page before undergoing orthodontic treatment.

Despite the above, relatively few studies have analyzed whether the use of dental practice social media by patients influences their decision to change practice. Most studies have analyzed the factors that influence patients when choosing a dental practice [29], the use of dental practice social media [23], opinions regarding dental practice social media [30], and the factors patients consider important when selecting a dental practice [23, 30]. No study, however, has examined whether patients make use of dental practice social media before changing dental practice, and whether such usage influences the eventual decision to change.

The goal of this study was to examine the influence of the use of dental practice social media on patients when last they changed their provider of dental care, and whether gender made any difference in this respect. The specific objectives were (1) to analyze the use of social media made by patients when changing dental practice, and (2) to determine the factors considered important when choosing a dental practice.

## Methods

### Recruitment

This cross-sectional study involved the use of a web-based questionnaire. The minimum required sample size (385) was calculated based on a confidence interval of 95% and a margin of error of 5%. All participants were required to be at least 18 years of age, live in Spain, and to make use of dental services and social media. Dentists and dental students were excluded, as were people whose behavior, or the factors they might consider important, was potentially different to that of the general public.

Participants were recruited and responded (on their own devices) to the questionnaire via the authors' social networks: WhatsApp (Y.F.; A.S.; V.DF.G.), LinkedIn (Y.F.), and Facebook (A.S., G.J., M.G.). Responses were also collected using a tablet provided at the Villaviciosa de Odón campus of the Universidad Europea de Madrid. The questionnaire was made available from February 25th to June 25th, 2022.

### Ethics approval

This study was approved by the Ethics Committee of the Universidad Europea de Madrid (Nº. CIPI/22.022). All participants were informed about the objectives of the study and the protection of the privacy and confidentiality of their data in accordance with regulations (EU) 2016/679 of the European Parliament, and of the European Council 27th April 2016, regarding the protection of personal data, its processing and free movement. All data were collected anonymously. Participants were informed that they could leave the study at any time. All participants gave their informed consent regarding inclusion. No compensation was provided for participation.

### Questionnaire

Microsoft Forms (Microsoft Inc., Seattle, WA, USA) was used to design the study questionnaire since no registration is required and the service is accessible via different electronic devices (tablets, smartphones, laptops). For validation purposes, a pilot version of the questionnaire was prepared, in Spanish, consisting of 21 questions in four sections (estimated completion time around 3 min):

#### Informed consent

In this first section, the objectives of the study were presented, the principal investigator (V.DF.G.) was identified, and participants were informed of the duration of the survey, the voluntary and anonymous nature of their participation, and the option to withdraw at any time. Consent was obtained through a dichotomous question (yes/no). Those who chose not to provide informed consent did not proceed with the survey.

#### Sociodemographic characteristics

Sociodemographic information was collected via four questions. Two were dichotomous, asking whether the respondent was a dentist or dental student, and whether the respondent was male or female. The other two were polytomous, asking for information on age group (18–25; 26–35; 36–45; 46–60; > 61) and the last time the respondent had changed dental practice (this year or last year; 2 to 5 years ago; 6 to 10 years ago; > 11 years ago).

#### Patient behavior

This section collected information on attitudes towards dental practice social media and consisted of the questions below. For the design of the first four, published articles on patient behavior regarding the use of dental practice social media [23, 30] were used to help adapt them to the objectives of the study.

Have you ever visited the website or social media of a dental practice? If the response was 'yes', the respondent was asked to:

- Please indicate the platform/social media you visited (response: Website/Instagram/Facebook/YouTube/Twitter/LinkedIn)
- Please indicate the main reason you make use of your dental practice's social media (response: educational purposes/to know the facilities of the dental practice/to meet the team/to see the prices of the treatments/to look for promotions)

Do you follow your dental practice on social media? If the response was 'yes', the respondent was asked to:

- Please indicate the platform/social media you use (response: Website/Instagram/Facebook/YouTube/Twitter/LinkedIn)
- Please indicate the main reason you follow your dental practice (response: educational purposes/to know the facilities of the dental practice/to meet the team/to see the prices of the treatments/to look for promotions)

Before changing dental practice, did you check the Internet/dental practice social media (Website, Facebook, YouTube, Instagram, LinkedIn, Twitter)? If the response was 'yes', the respondent was asked to:

- Please indicate the platform/social media you used (response: Website/Instagram/Facebook/YouTube/Twitter/LinkedIn)

Did visiting dental practice social media influence your decision when changing dental practice? If the response was 'yes', the respondent was asked to:

- Please indicate the platform/social media you checked before choosing a new dental practice (response: Website/Instagram/Facebook/YouTube/Twitter/LinkedIn).

#### Factors that respondents considered important when choosing a dental practice

Information on factors that respondents considered most important when choosing dental practice was collected via six questions on a 3-point Likert scale (not important, important, very important). The questions in the fourth section used those previously reported by other researchers [23, 30]. The factors focused on were: presence on social media, website quality, online reviews, reimbursement of treatment costs, facilities and technologies, and recommendations from friends/family.

The questions in all four sections were evaluated by 10 university professors. They were asked whether they would include the questions (yes/no), and to indicate the importance of each on a 5-point Likert scale (very important, important, neutral, low importance, not important). This led to the questions ‘Please indicate the main reason you make use of, and follow, your dental practice's social media' being discarded. Finally, 10 dental students were requested to indicate the clarity of the remaining 19 questions on a 5-point Likert scale (strongly agree, agree, neutral, disagree, strongly disagree). All questions were considered clear. The final questionnaire consisted of these 19 questions.

### Statistical analysis

All survey data were collected and stored in a password-protected Excel spreadsheet (Microsoft, Albuquerque, New Mexico, USA) to which only the principal investigator had access. The survey was then deleted to prevent any potential security issues. The sociodemographic characteristics of the respondents, and the values recorded for the variables of interest, were subjected to descriptive analysis. The Pearson Chi-squared test was used to analyze the relationship between variables. Significance was set at *p* > 0.05. All calculations were made using SPSS software (IBM SPSS Statistics, v.22, 2013).

## Results

### Sample characteristics

The questionnaire was seen by 155 potential respondents via WhatsApp, by 504 via LinkedIn, by 118 via Facebook, and by 51 via the supplied tablet (total = 828). The response rate was 71.01% (588/828) and the questionnaire completion rate 99.65% (586/588). Ten respondents did not provide informed consent, 26 were under 18, and 49 were dentists/dental students; with their exclusion, a total of 503 responses were included in the final analysis.

Table 1 summarizes the sex, age, and the last time the respondents changed dental practice. 62%, (312/503) of respondents were female. Most participants changed dental practice either within the last 2–5 years (30%, 151/503) or more than 11 years ago (29.6%, 149/503).

**Table 1:** Respondent sociodemographic data (*N* = 503)

Characteristic	Participants
**Sex, n (%)**	
Male	191 (38)
Female	312 (62)
**Age group (years), n (%)**	
18–25	106 (21.1)
26–35	119 (23.7)
36–45	103 (20.5)
46–60	108 (21.5)
> 61	67 (13.2)
**Last change of dental practice, n (%)**	
This or last year	113 (22.5)
2 to 5 years ago	151 (30)
6 to 10 years ago	90 (17.9)
More than 11 years ago	149 (29.6)

### Respondent behavior with respect to dental practice social media

Table 2 shows the respondents' behavior in relation to dental practice social media. 41.4% (208/503) of the participants indicated that they had interacted with these media, and 7.2% (36/503) that they followed their dental practice. The website was the most frequently visited platform, although Instagram was that most used to follow a dental practice.

**Table 2:** Respondent behavior in relation to dental practice social media (*N* = 503)

Characteristic	Respondents
**Participants that visited dental practice social media, n (%)**	
Yes	208 (41.4)
No	295 (58.6)
**Platform used**^**a**^**, n (%)**	
Facebook	38 (18.3)
Instagram	54 (26)
LinkedIn	10 (4.8)
Twitter	8 (3.9)
Website	173 (83.2)
YouTube	15 (7.2)
**Participants that followed dental practice social media, n (%)**	
Yes	36 (7.2)
No	467 (92.8)
**Platform on which followed**^**a**^**, n (%)**	
Facebook	15 (41.7)
Instagram	23 (63.9)
LinkedIn	3 (8.3)
Twitter	4 (11.1)
Website	7 (19.4)
YouTube	2 (5.6)

^a^Given that respondents could indicate several platforms, the sum of the percentage values is not 100

Table 3 shows that 23.5% (118/503) of the respondents made use of dental practice social media before changing dental practice. Further, 86.4% (102/118) of these indicated that their decision to change was influenced by the information obtained (Table 4). The most used platform in this regard was the dental practice website.

**Table 3:** Respondent behavior in relation to the use of dental practice social media before changing dental practice (*N* = 503)

Characteristic	Respondents
**Respondents that visited dental practice social media before changing dental practice, n (%)**	
Yes	118 (23.5)
No	395 (76.5)
**Platform visited**^**a**^	
Facebook	16 (13.6)
Instagram	21 (17.8)
LinkedIn	5 (4.2)
Twitter	6 (5.1)
Website	100 (84.7)
YouTube	1 (0.8)

^a^Given that respondents could indicate several platforms, the sum of the percentage values is not 100

**Table 4:** Influenced of dental practice social media on the decision to change practice (*n* = 118)

Characteristic	Respondents
**Participants that were influenced by visiting dental practice social media before changing dental practice, n (%)**	
Yes	102 (86.4)
No	16 (13.6)
**Platform visited**^**a**^**, n (%)**	
Facebook	12 (11.8)
Instagram	19 (18.6)
LinkedIn	4 (3.9)
Twitter	5 (4.9)
Website	87 (85.3)
YouTube	1 (1)

^a^Given that respondents could indicate several platforms, the sum of the percentage values is not 100

A relationship was detected between the last time the respondents changed dental practice and the use made of dental practice social media. The respondents who changed dental practice under 5 years ago, or in the present/past year, more often consulted these media than did those who changed dental practice over 11 years ago (*p* > 0.05) (Table 5). No differences were observed between males and female (*p* > 0.05) (Table 5).

**Table 5:** Use of dental practice social media before changing dental practice according to sex and time of last changing dental practice (*n* = 118)

Variables		
	Respondents, n (%)	*p-*value
**Sex**		
Male	46 (39)	*p* > .05
Female	72 (61)	*p* > .05
**Last time dental practice was changed**		
This or last year	36 (30.5)	*p* < .05
2 to 5 years ago	53 (45)	*p* < .05
6 to 10 years ago	21 (17.8)	*p* > .05
More than 11 years ago	8 (6.7)	*p* < .05

No differences were observed between males and female in terms of the influence of dental practice social media on the decision to change dental practice (*p* > 0.05). The respondents who changed dental practice in the present/past year were more often influenced to do so by dental practice social media than were any other respondents (*p* < 0.05) (Table 6).

**Table 6:** Influence of social media on the decision to change dental practice according to sex and time of last changing dental practice (*n* = 102)

Variable		
	Participants, n (%)	*p-*value
**Sex**		
Male	40 (39.2)	*p* > .05
Female	62 (60.8)	*p* > .05
**Influence on the decision visiting social media**		
This or last year	34 (33.3)	*p* < .05
2 to 5 years ago	45 (44.1)	*p* > .05
6 to 10 years ago	16 (15.7)	*p* > .05
More than 11 years ago	7 (6.9)	*p* > .05

### Factors deemed important when choosing a dental practice

**Fig. 1:**
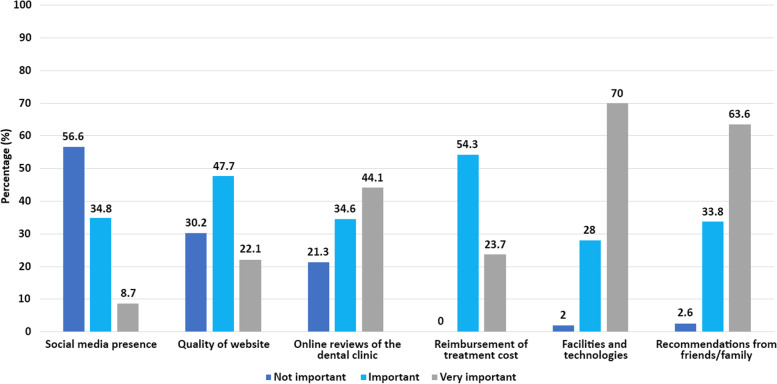
Factors deemed important when choosing a dental practice

‘Facilities and technology’ was the most valued factor in the decision to choose a dental practice followed by 'recommendation from friends and family'. The least valued factors were social media presence and website quality. No significant differences were observed between males and females in this respect (*p* > 0.05) (Fig. 1).

## Discussion

Recent years have seen a huge increase in access to the Internet and the use of social media [5]. Social media are also in use in the dentistry sector, with possible benefits for dental practices [31]. Most patients are users of social media [5, 7]. In the present study, almost half of respondents (208/503; 41.1%) had made use of dental practice social media; this agrees with results from a UK study [23]. However, the percentage of users who did so was lower than the total percentage of individuals who made general use of social media [5]. This lower interest was also reflected in the low percentage of respondents (36/503; 7.2%) who indicated having followed a dental practice. This finding is in line with that reported by Parmar et al. [23], who found that less than half of patients who searched for their dentist on social media had added him or her as a 'friend'.

Previous studies have shown that, among dental practices, the main reason for using social media was marketing [25]. Indeed, dentists have been reported to consider social media as a marketing tool [23]. The present work analyzed whether patients used social media before changing dental practice, and only 23.5% (118/503) indicated that they had. This shows that when patients choose a dental practice, other factors play an important role, possibly including the dentist's reputation, the characteristics of the dentist, recommendation from friends and family, or technology and facilities [23, 29–31]. However, among those who did make use of dental practice social media before switching, the majority (102/118; 86.4%) indicated that this had influenced their decision. Other authors report potentially interested patients might be positively influenced by dental practice social media in their decision to proceed to treatment [1]. Thus, despite the percentage of patients who made use of these media before changing their dental practice being low, their influence on patients who did use them was high. Dentists might be advised to regard these platforms as communication and marketing tools that might bring them new patients.

No differences were seen between males and females either in their use of dental practice social media before changing dental practice, or in the influence of these media on that decision. This is in line with that reported by Parmar et al. [23]. However, other studies have reported females to show a preference towards practices that use social media [30, 31], while others describe more males to seek information about dental practices on social media [29]. Some authors indicate that females more often follow their dentists on social media [32].

The results also show that those who changed dental practice in the last 5 years made use of dental practice social media more often than did those who changed practice over 6 years ago. In addition, the use of these media had a greater influence on the decision made by the respondents who changed dental practice in the present/past year. This suggests that, coinciding with wider access to the Internet and the greater use of social media [5, 17, 30], more patients are now using these platforms before changing dental practice.

Practice websites were the most used source of information and certainly the most consulted before changing dental practice. However, Instagram and Facebook were the most used to follow the practice. The agrees with Cox et al. [7] who reported that the likelihood of visiting a practice's website before undergoing treatment was greater than the likelihood of visiting its Facebook page. However, Henry et al. [25] reported that Facebook was the platform most visited.

The use of social media has increased significantly in recent years [33]. Therefore, it is relevant to investigate its relationship with dental clinics. However, currently there are other factors, such as facilities and technology, and recommendations from friends and family, that patients prioritize when selecting a dental clinic, as indicated by other authors [23, 30].

This study suffers from the limitation that it was conducted in only one country, and the use of the Internet and/or social media differs from country to country [5]. The work should therefore be repeated elsewhere. In addition, the > 61 age group was composed of fewer respondents than the other groups, possibly related to the lesser use of the Internet by older people [34]. Furthermore, the possibility exists that a single user could have completed the survey multiple times [35]. When using Microsoft Forms, it is difficult to prevent this. However, in a previous study [36] only 0.9% (4/458) of respondents were found to have done so.

Future studies could evaluate the use of social media by patients as an educational tool, for scheduling appointments, or for communicating with dental professionals.

## Conclusions

Some 41.1% (208/503) of patients indicated that they had made use of dental practice social media. Before changing dental practice, 23.5% (118/503) of respondents had done so, and 86.4% of these (102/118) indicated that this had influenced their decision to change. Patients who had changed practice within the last 5 years used dental practice social media more often. In addition, those who had changed in the present/past year reported that the use of these media had significantly influenced their decision to change. No significant differences were seen between males and females with respect to any measured variable. Although there are other factors that influence the selection of a dental practice, in recent years the social media of clinics have become more important. Dentists might therefore do well to consider social media as communication and marketing tools.

## Data Availability

The datasets generated for this study are available from the corresponding author upon reasonable request.
